# Reid on olfaction and secondary qualities

**DOI:** 10.3389/fpsyg.2013.00974

**Published:** 2013-12-24

**Authors:** Jake Quilty-Dunn

**Affiliations:** Philosophy and Cognitive Science, CUNY Graduate CenterNew York, NY, USA

**Keywords:** reid, olfaction, perceptual learning, sensation and perception, philosophy of perception

## Abstract

Thomas Reid is one of the primary early expositors of the “dual-component” theory of perception, according to which conscious perception constitutively involves a non-intentional sensation accompanied by a noninferential perceptual belief. In this paper, I will explore Reid's account of olfactory perception, and of odor as a secondary quality. Reid is often taken to endorse a broadly Lockean picture of secondary qualities, according to which they are simply dispositions to cause sensations. This picture creates problems, however, for Reid's account of how we perceive secondary qualities, including odors. Given Reid's insistence that we come to be aware of odors only by inferring a causal relation to obtain between them and our olfactory sensations, it seems that he cannot allow for direct, noninferential perceptual awareness of odors. Since his general account of perception invokes noninferential perceptual beliefs to explain perceptual awareness, it seems that Reid must either reject this general account for the case of olfactory perception (and supplant it with something else), or else deny that we ever actually perceive odors. I will attempt to reconcile these ideas by appeal to Reid's doctrine of “acquired perception,” which involves the incorporation of learned conceptual representations into perceptual states via perceptual learning. Reidian acquired perception enables genuine olfactory perceptual acquaintance with odors despite the dependence of the semantic properties of the relevant representations on causal relations to sensations. In exploring these issues, I hope to illuminate several features of Reid's account of perception and demonstrate its interest to contemporary theorizing about conscious perception—especially olfaction—in the process. Reid's theory of olfaction remains a live, coherent option for present-day theorists.

## “Of smelling”

In a letter to Hugh Blair, dated 4 July 1762, David Hume commented on the manuscript of Thomas Reid's first major philosophical work, *An inquiry into the human mind on the principles of common sense* (‘*IHM*’). Hume complimented its literary qualities, but noted that “there seems to be some Defect in Method” (*IHM* Appendix 1.1, 256). “For instance,” Hume offered, “under the Article of Smelling, he gives you a Glimpse of all the Depths of his Philosophy” (*Ibid*.).

Reid took a thorough investigation of perception to be fundamental to a proper philosophical understanding of how the mind works. Besides the introduction and conclusion, each chapter of *IHM* is devoted to one of the five senses. Reasoning that it “is so difficult to unravel the operations of the human understanding” that we cannot expect to succeed except by “beginning with the simplest, and proceeding by very cautious steps to the most complex,” Reid begins with a chapter on olfaction, “Of smelling” (*IHM* 2.1, 25). As Hume noted, the chapter is rich and disparate in its contents; it is larger than the chapters on gustation and audition combined, and is nearly as long as the chapter on tactition (the chapter on vision, perhaps unsurprisingly, dwarfs them all).

In this paper, I will explore Reid's theory of olfactory perception as a special—and especially pure—case of his theory of the perception of secondary qualities generally. His theory of secondary-quality perception, including olfaction, appears to have serious problems. My aim by the end of the paper is to provide an understanding of Reid's account of olfactory perception (and secondary-quality perception in general) that does justice to his general theory of perception and his notion of odors^1^ and the other secondary qualities^2^. Hume's remark is apt; understanding Reid's theory of olfaction will require calling upon many other aspects of Reid's theory of perception and his general philosophy of mind.

The following discussion is historical in nature, but it will be valuable to many who are interested in olfactory perception, and the perception of secondary-qualities generally. As will be explained below, problems for Reid's account arise from the conjunction of two theses that many hold today: that perception is partly a matter of noninferential intentional awareness of qualities of external objects, and that odors are dispositions (or the bases of dispositions) to cause sensations. If the discussion below is correct, then these theses can be comfortably reconciled, which should be of interest to contemporary theorists. Furthermore, I will explore Reid's theory of “acquired” perception, which occurs when learned contents are incorporated into perceptual experience. This controversial idea is also part of contemporary philosophical discussion (see, for example, Churchland, 1979), and solving problems that arise for Reid's account will aid in understanding the nature of acquired perception. Finally, I will explain how Reidian olfactory perception accommodates both the qualitative and representational aspects of olfactory perceptual experience, while making minimal ontological commitments about the nature of odor. Clarifying and understanding Reid's theory of olfactory perception sheds light on all these contemporary issues, and provides a coherent and arguably attractive account of olfaction.

## Setting up the problem

### Perception

Reid thinks the senses each deserve their own investigation, but he does have a general outline of how perception works, which Wolterstorff (2001) calls the Standard Schema. According to the schema, perception is a matter of having a sensation, which has no intentional content and is individuated by its felt quality, and then noninferentially forming a “conception and belief” of an external object and/or its properties, relations, and so on. Similar views of perception crop up quite frequently throughout the history of philosophy. The idea that perceptual awareness of outer objects is a matter of sensations giving rise to conceptual representations is perhaps most often identified with Kant. It was endorsed in the 20th century by Sellars (1956), and it (or something very close to it) is held by his various philosophical inheritors, e.g., Churchland (1979); Rosenthal (2005), and Coates (2007). Though Reid often receives credit for the origination of the view and the sharp sensation–perception distinction it licenses, A. D. Smith, who refers to the theory as the *dual-component view*, claims to find it earlier in the work of Malebranche, Digby, and sargeant (Smith, 2002, 284n17). Kuehn (1987) makes a strong historical case that the similarities between Reid and Kant are in fact due to a direct, as well as indirect, Reidian influence on Kant's thought.

It is crucial for Reid's general picture of perception that the intentional component of perception (the conception and belief)^3^ make one immediately or directly aware of external objects and their properties. One of Reid's stated purposes in philosophy is to avoid succumbing to the theory of ideas, according to which (as Reid understands it) perceiving external objects is a matter of directly and noninferentially perceiving some mental entity—an idea, in Locke's and Berkeley's terminology, or an impression in Hume's—and positing a relation of some kind to obtain between the mental entity and some object in the world, or else leaving out the external world altogether. Reid took Berkeley and Hume to infer their radical epistemological and metaphysical conclusions from this premise, and thought their inferences valid. He saw this as a good sign that the premise was likely false, and tried to build another account of perception and knowledge that would explain the relevant phenomena equally well, and without what he saw as highly implausible conclusions^4^.

Reid's account of perceptual intentionality is direct in two senses that are relevant to the ensuing discussion. First, the intentional component of perception is noninferential. It does not arise out of any process that could reasonably be called inference, and in that respect it constitutes a direct awareness of the object represented. Second, perceptual intentionality is, to borrow a phrase from Tyler Burge, “referentially non-derivative” (Burge, 2005, p. 30). That is, it does not refer to its object in virtue of referring to something else. In Acquired Acquaintance, I will place more restrictive conditions on perceptual awareness, but for now, these two ways in which, for Reid, the intentional component of perception is direct will suffice.

### Secondary qualities

One of the few things Reid agrees with Locke about is the legitimacy of the distinction between primary and secondary qualities. He does not agree, however, with Locke's way of drawing the distinction. For Locke, the mental states that stand for primary qualities “are resemblances of them,” whereas ideas of secondary qualities “have no resemblance of them at all” (Locke, 1690, p. 2.8.15, 137). Reid, conversely, does not think mental states can ever resemble qualities of external objects, so neither primary nor secondary qualities resemble our sensations or perceptions of them (see *IHM* 5.8, 75; Van Cleve, 2011); to think otherwise, for Reid, is to make a basic category error that reveals deep philosophical confusion. For Reid, the distinction between primary and secondary qualities has to do, at least in part, with the kinds of understanding we have of different qualities of objects. With respect to the primary qualities, we have “a direct and distinct notion,” but of secondary qualities “only a relative notion, which must, because it is only relative, be obscure” (*EIP* 2.17, 202). Our notions of secondary qualities are relative because such qualities “are conceived only as the unknown causes or occasions of certain sensations with which we are well acquainted” (*Ibid*.); because the causes are unknown, they are obscure to us. There is an interesting debate in the secondary literature on Reid as to whether his ontological account of secondary qualities has them as dispositions to cause sensations or as the bases of those dispositions^5^. In either case, however, it is clear that we only really think there are secondary qualities at all in virtue of positing a causal relation to obtain between them and our sensations, and that our ordinary knowledge of them does not progress beyond that relative and obscure notion.

### The problem

Perhaps the problem is already clear. On the one hand, Reid says perceptual intentionality is not based on inference, and refers to its objects non-derivatively, i.e., not in virtue of referring to anything else. On the other hand, Reid says our notions of secondary qualities like odors are not direct, and are ostensibly based on our inferring there to be external qualities that cause our sensations. Our awareness of secondary qualities, such as it is, appears therefore to be both inferential and referentially dependent on sensations. There has been considerable controversy about whether Reidian sensations' mediating perceptual intentionality, as occurs even in the perception of primary qualities, renders Reid's general view of perception indirect in some Lockean sense^6^. I am not interested in that question here, however. Given that the very content of our notion of a secondary quality such as odor is dependent on an inference from awareness of sensations, for Reid, it seems impossible that we could ever actually perceive odors or any secondary qualities at all on his view. This is the conundrum. Van Cleve (in press) argues that the proposition that perception is immediate, the proposition that our notions of secondary qualities are relative and obscure, and the proposition that we can perceive secondary qualities, form an inconsistent triad.

## Two solutions

### Original and acquired perception

I will sketch two possible solutions to the problem just raised, one that relies on nativism (which Reid generally endorsed), and one that relies on perceptual learning. First, it will be helpful to explain a crucial aspect of Reid's philosophy, namely, the distinction between *original* and *acquired* perception. As mentioned in the previous section, Reidian perception occurs when sensations give rise to noninferential intentional states that take external things as their objects. It is an open question to what extent the patterns of mental causation that relate sensations to perceptual intentional states are learned, and to what extent they are innate. Wilfrid Sellars, who seems to endorse the same broad view of perceptual awareness^7^, argues that the very capacity to have intentional states is entirely learned, and so he would deny that the relevant causal connections between the sensory and intentional components of perception are innate to any extent (Sellars, 1956). Reid, on the other hand, endorses a strong form of nativism, according to which certain kinds of sensations give rise to certain kinds of intentional states due to the nature “of our constitution” (see, e.g., *IHM* 5.2, 56). Reid calls this sort of perception “original perception” (e.g., *IHM* 6.21, 177), and contrasts it with “acquired perception,” which occurs when the causal connections between the sensory and the intentional components of perception are acquired through habituation (e.g., *IHM* 6.21, 177–178).

There are some uncontroversial examples of qualities that are perceived through original perception, but they are few in number, and are, somewhat surprisingly, mostly proprietary to tactition. They include tactile perception of texture, solidity, shape, motion, and what Reid calls “hardness,” which is the propensity of a body to resist deformation in response to pressure (*IHM*, Chapter 5). They also include the visual perception of what Reid calls the “visible figures” of objects (*IHM* 6.22, 186), which are two-dimensional forms that operate according to a non-Euclidean spherical geometry (*IHM* 6.9)^8^. Uncontroversial examples of acquired perception include the visual perception of three-dimensional figure (which Reid calls “real figure,” and also, because it is originally perceived only through tactition, “tangible figure”). They also include perceiving what are today called higher-level properties, or as Siegel (2011) calls them, “K-properties.” According to Reid, of all our perceptual capacities, “the far greater part is *acquired*, and the fruit of experience” (*EIP* 2.21, 235; italics his).

The farmer perceives by his eye, very nearly, the quantity of hay in a rick, or of corn in a heap. The sailor sees the burthen, the built, and the distance of a ship at sea, while she is a great way off. Every man accustomed to writing, distinguishes his acquaintance by their hand-writing, as he does by their faces. And the painter distinguishes in the works of his art, the style of all the great masters. In a word, acquired perception is very different in different persons, according to the diversity of the objects about which they are employed, and the application they bestow in observing them.

(*IHM* 6.20, 172)

It is not obvious how olfactory perception fits into the original–acquired dichotomy. I will describe two ways of solving the problem of olfactory perception, one according to which olfactory perception is acquired, and another according to which odors are perceived originally. The acquired story is superior, though it will require some work to make sense of it.

### Acquired olfactory perception

By far the more popular solution in the secondary literature is to interpret Reid as saying that secondary qualities are perceived only via acquired perception. This approach is endorsed by, at least, Lehrer (1978), McKitrick (2002), Nichols (2007), Buras (2009). As McKitrick says, we “do not originally perceive secondary qualities, except as unknown causes of sensations. It is not a part of our original constitution that sensations produced by secondary qualities give us perceptions of those qualities as they are in themselves” (McKitrick, 2002, p. 493). On the contrary, according to this interpretation, our conception of secondary qualities is in the first instance a theoretical inference from our sensations to their causes. We can only come to perceive secondary qualities by incorporating this conception into occurrent perceptions. Since Reid says “our senses give us only a relative and indirect notion” of secondary qualities (*EIP* 2.17, 201), it seems natural to say that, in terms of original perception, we do not have any sort of direct perceptual awareness of odors. Acquired perception would thus be required to supplement our endowed perceptual capacities.

The problem with this route is that it does not seem immediately to avoid the crucial problem of the perception of secondary qualities. The problem, recall, is this: if our conception of secondary qualities is an inference from our sensations to some unknown outer cause, and if perceptual intentionality must be noninferential and referentially non-derivative, then we cannot perceive secondary qualities. McKitrick simply says that such perception is “mediated” by our awareness of sensations (McKitrick, 2002, p. 494; see also Nichols, 2007, p. 169)—but of course, by Reid's measure of what constitutes perception, such “perception” is not really perception. It is just an inference based on awareness of a mental entity, e.g., an olfactory sensation. According to Nichols, “our perceptual beliefs about primary qualities conform to a non−inferential theory” of perceptual knowledge, while those about secondary qualities do not (Nichols, 2007, p. 215).

There is a way out of this problem. It is crucial to distinguish between a given notion's referring to type A by means of reference to a relation to type B, and a token intentional state's referring to a token of A by means of a token of B. Less abstractly, there is a difference between our notion of a given type of odor, O, having the content, The kind of property that causes sensations of type O^*^, and my occurrent awareness of the instance of O in my environment being referentially derivative of my awareness of my token sensation of O^*^^9^. These two ideas are doubly dissociable. On the one hand, my notion of rain is not referentially or inferentially dependent on my notion of a lawn chair cushion's being wet, but my occurrent belief that it rained might be based on an inference from the wetness of the cushion. On the other hand, and more importantly for the case of olfactory perception, my notion of what it is to be an odor of type O might be wholly dependent on O's bearing certain relations to sensations, but my occurrent perceptual belief that there is an instance of O before me need not be an inference from my awareness of a token O^*^ sensation. This latter case is enabled by the possibility of acquired perception, whereby conceptions formed initially through inference or some other non-innate procedure can become noninferential constituents of perceptual intentional states. For instance, where I might once have thought there was a car in the street because I thought a car was the likely cause of my sensations, through perceptual learning, I gain the capacity to form, noninferentially, an auditory perception as of a car in the street^10^, ^11^.

The case is confused with olfaction (and other secondary qualities) because the semantics of our conception of a given odor O involves a description of a relation it stands in to sensations. That seems to imply that becoming aware of O in perception is really just a matter of introspectively becoming aware of sensations and positing such a relation to obtain between *my present sensations* and some odor in the environment. But that simply does not follow. If sensations can noninferentially give rise to conceptions, then a sensation of type O^*^ could noninferentially give rise to a conception of O; in that case, my awareness of O will not be an inference from O^*^. It will just also happen to be the case, given the vagaries of olfaction, that my notion of what O is depends on its relations to O^*^, and my acquisition of that notion *did* depend on inference from O^*^ sensations.

The semantics of our conception of odor can be derivative from our awareness of sensations without making every instance of our perceptual awareness of odors being so derivative. The primary function of acquired perception, for Reid, is to enable inferential awareness to transform into noninferential perception. The particularly indirect and relative semantics of our conceptions of olfactory properties obscures this point in the case of olfactory perception, but there as elsewhere, our indirect conceptions can become incorporated into perceptual awareness, thus yielding direct perception of odors in the environment without being mediated by occurrent awareness of token sensations. Though the semantics of our conception of O is referentially derivative at the level of types—that is, our conception refers to odors of type O via referring to a relation that obtains between such odors and sensations of type O^*^—the token representation of O that figures in an acquired olfactory perception would be referentially non-derivative at the token level, since it does not refer to the token instance of O in the environment in virtue of referring to a token O^*^ sensation. No token acquired perception, therefore, is referentially derivative of any token sensation, even if the type of which the perceptual representation is a token is referentially derivative of sensation-types.

The acquisition of our conceptions of odors would, in this story, be causally dependent on our already being able to form conceptions of objects, perhaps via original perceptions of their primary qualities. We may, in the first instance, be perceptually aware of objects by means of their primary qualities and also be aware of our olfactory sensations; we may then posit some unknown quality in the objects we perceive that is causally responsible for our olfactory sensations. Through a gradual trial-and-error learning process, we form fine-grained conceptions of odors, and through the process of perceptual learning, we become able to trigger these conceptions in acquired perceptions without undergoing any inference or making reference to our occurrent olfactory sensations. A passage where Reid discusses the acquisition of our conceptions of colors provides a helpful analog:

By the constitution of nature, we are led to conceive this [color sensation] as a sign of something external. A thousand experiments for this purpose are made each day by children, even before they come to the use of reason. They look at things, they handle them, they put them in various positions, at different distances, and in different lights. The [sensations] of sight, by these means, become associated with, and readily to suggest, things external, and altogether unlike them. In particular, that [sensation] which we have called the appearance of color, suggests the conception and belief of some unknown quality, which occasions the idea; and it is to this quality, and not to the [sensation], that we give the name of color.

(*IHM* 6.4, 86)

### Original olfactory perception?

One could argue that there is original perception of some secondary qualities. Van Cleve suggests that perhaps original perception of secondary qualities can be said to occur if it allows for the subject to locate the instance of the quality in space. As he observes, however, this would rule out olfaction if we “have no such innate ability to localize the causes of our olfactory sensations” (Van Cleve, in press).

Reid seems to say, however, that though olfaction and audition do not enable original localization, they still involve original perception:

In smelling, and in hearing, we have a sensation or impression upon the mind, which, by our constitution, we conceive to be a sign of something external: but the position of this external thing, with regard to the organ of sense, is not presented to the mind along with the sensation.

(*IHM* 6.8, 99)

Van Cleve suggests that we may “have an original perception to the effect that *some* quality exists that is causing our sensation or, more colloquially, that a certain scent is in the air,”^12^ and that “one could even be said to have an objectual perception of the scent or quality itself, without knowing where it resides” (Van Cleve, in press)^13^.

The question would immediately arise, of course, as to whether the awareness we come to form of the odor is actually just an inference from our awareness of the sensation. Reid seems to talk as though it is inferential when he says that the senses originally give us only a relative and indirect notion of odor. Perhaps, on the other hand, the account could work in the exact opposite direction from the acquired-perception account. That is, instead of saying we gain a notion of odor through inferences from sensations, and then incorporate it into perception through perceptual learning, yielding noninferential perceptual awareness of odors, we could say that we *begin* with such noninferential perceptual awareness of odors, and through exploring what our notion of an odor is, develop an indirect and relative notion. This story is undeveloped as it stands, but there is nothing internally inconsistent in it.

Nonetheless, the nativist story leaves the origin of our capacity to represent odors noninferentially unexplained, whereas the acquired-perception story explains it in terms of our inferentially taking there to be causes of our sensations and eventually coming to represent such causes noninferentially without referring to token sensations. Indeed, though the passage quoted above in this section does speak of “our constitution” and thus suggests original perception, it could be that Reid simply means we have a natural tendency to posit causes of our sensations, as he says children theorize about the causes of color sensations. The acquired account is preferable on the theoretical grounds that it enables a fuller explanation of how olfactory perception takes place, and there does not appear to be textual evidence that cuts unambiguously against it. Furthermore, Reid's criteria for positing innate psychological laws include the inability to explain the relevant psychological phenomena “by tradition, by education, or by experience” (*IHM* 5.2, 58). The mere existence of a coherent and explanatorily efficacious account of olfaction in terms of perceptual learning would thus suffice by Reid's own lights to make a nativist explanation unnecessary. There are serious (though surmountable) problems for the acquired account, however, to which I turn now.

## Acquired acquaintance

### Acquired perception not perception?

One might be inclined to challenge the idea that acquired perception is actually *perception*. The argument arises because lots of cases that seem to fit the criteria for acquired perception do not seem like perception. For van Cleve, for example, one's awareness of the external environment should only be considered perceptual awareness if it involves “conception of the acquaintance variety” (Van Cleve, 2004; in press), which he argues acquired perception does not involve. As noted above, perception, according to Reid, involves “conception and belief.” Conception is simply the intentionality-providing component of all intentional mental states; it may therefore be open to further debate whether it involves conceptual representation in something like the contemporary sense (Alston, 1989). Of course, Reid does always talk of conception in perception as paired with belief, perhaps suggesting that it is a form of intentional content that can figure in a belief (and thus, perhaps trivially, that it is conceptual in nature). However, he also talks of conception occurring by itself, in cases of “simple apprehension,” which for Reid is merely having a thing in mind, without predicating anything of it such that one's mental state is truth-evaluable (*EIP* 1.7, 65). It thus seems possible to construe different kinds of conception as involving different sorts of contents (Alston, 1989). One might, therefore, argue that what distinguishes perception from mere belief is the kind of conception or awareness involved, perhaps in terms of nonconceptual content (e.g., Copenhaver, 2010; Quilty-Dunn, 2013).

The immediate question is, what forms of awareness constitute acquaintance? Perhaps we can characterize perceptual acquaintance without taking a stand on the kind of content involved in the relevant state^14^. The term “acquaintance” evokes a heavy load of Russellian baggage—van Cleve says he means it “in something like Russell's sense” (Van Cleve, in press). In order to understand the proposal better, I will turn briefly to Russell's notion of acquaintance. Russell says, “I am acquainted with an object when I have a direct cognitive relation to that object, i.e., when I am directly aware of the object itself” (Russell, 1910, p. 108). For Russell, of course, the objects with which we are directly related must be sense-data, and cannot be ordinary outer objects. When discussing the perception of external objects and their qualities, then, we can put that assumption aside. Russell also attaches unique epistemic significance to acquaintance. When one is acquainted with an object, “no further knowledge of it itself is even theoretically possible” (Russell, 1912, Ch. 5, 32). Again, this aspect of Russellian acquaintance simply could not extend to the perception of external objects. While Reid would certainly say that knowledge of objects gained through perception occupies a special epistemic position, there is little reason to saddle Reid with the false view that perceptual knowledge is unimprovable, since we could always get a better view on an external object, and gain further, more accurate, and more complete knowledge of it and its properties. Relatedly, one could (along Russell's lines) endorse perceptual acquaintance as intrinsically veridical, and so if there is no object, there could not be perceptual acquaintance at all. This view in contemporary philosophy of perception is called *disjunctivism* (see, e.g., Burge, 2005; Martin, 2006; Brewer, 2011). It seems unlikely that Reid held such a view. First of all, Reidian perception is a complex of sensation and belief, which seems quite different from the nonrepresentational relation posited by disjunctivists. Second, there is no textual evidence to my knowledge that would license attributing disjunctivism to Reid. If an account of perceptual acquaintance can be constructed that does not involve a commitment to disjunctivism, it would therefore seem to be preferable.

Below, I will propose four conditions for perceptual acquaintance. I believe they capture the spirit of van Cleve's invocation of the Russellian notion, shorn of the baggage discharged in the previous paragraph. All four conditions can be applied to perceptual awareness of external objects (and not just sense-data), and indeed to Reidian acquired perception. These are not intended to be necessary or sufficient conditions. Rather, what follows is a sort of grab bag of properties that seem to mark many cases of perceptual acquaintance. The justification for appealing to these properties and not others is simply that they appear to characterize the cases that we would want to call perceptual acquaintance, and do not characterize cases that we wouldn't. The validity of these conditions should be judged on a case-by-case basis, to see whether they tend to apply where (and only where) we want them to.

### Conditions for perceptual acquaintance

First of all, acquaintance could be understood as involving *phenomenal immediacy*. By that I mean simply that our conscious awareness of the object is not preceded by a separate awareness of something else. This is presumably what Russell has in mind when he says one is “directly aware of the object itself” (Russell, 1910, p. 108). Since acquired perception involves noninferential perceptual beliefs that seem to the subject to be automatically activated and not to refer to their objects in virtue of referring to anything else, then the awareness they engender presents itself to the subject simply as an immediate awareness of a state of affairs in the environment.

Second, the relevant state could involve acquaintance if it is *psychologically noninferential*. This notion of acquaintance is similar to, though separate from, the point about phenomenal immediacy. Whereas that point has to do with whether the subject's conscious awareness of the object is manifestly derivative of her awareness of something else, psychological noninferentiality is simply a matter of the actual underlying psychological processes that give rise to the relevant intentional state. We can thus partially provide criteria for an intentional state's constituting acquaintance by stipulating that such states cannot arise through a psychological process of inference.

Third, acquaintance could be partially constituted by being *directly causally related* to the object. The object itself, and its qualities that are perceived, play a special and constitutive role in the causal process that brings about one's perceptual awareness. It is because the object is triangular that I represent it as triangular; I thus stand in a relation to it not merely of being accurately aware of its qualities, but also of its being responsible for my being so aware. Though one might take the acquaintance relation to be metaphysically thicker in some sense, it seems fair to say that the object's F-ness being directly causally responsible for one's veridical perception of its F-ness does justice to Russell's insistence on the acquaintance relation as one of “presentation” (Russell, 1910, p. 109). We can simply understand an object's presenting itself to us as a function of its causal efficacy in producing occurrent veridical perceptual awareness of it.

Fourth, acquaintance could be a matter of *sensory character*. This condition is a bit more difficult for Reid, who makes a sharp separation between the sensory component of perceptual awareness and the intentional component. Nonetheless, perceptual intentionality could be said to have a sensory character on a Reidian view insofar as the relevant intentional states are intimately tied to the qualitative character of sensations. Sensations and perceptual intentionality are, of course, metaphysically independent for Reid. There are still two important ways in which the qualitative character of sensations colors perceptual intentionality. On the one hand, sensations bear tight causal relations to the intentional components of perceptual awareness. Perceptual intentionality can thus be individuated from other forms of intentionality via its unique causal situation with respect to sensations. On the other hand, Reid often stresses that it is highly difficult, and perhaps sometimes impossible, introspectively to separate the relevant contributions to the felt character of a perceptual state. That is, from the first person, it is extremely unnatural and difficult to isolate the sensory component and the intentional component. Reidian perceptual experience presents itself to consciousness as a package deal, a unified sensory presentation of external objects and states of affairs. One might object to this last point that acquaintance is a first-order property of perception, and not a matter of the way in which one has a higher-order awareness of it. I do not see why this must be the case, however. There does not seem to be a principled reason to deny that an intentional state's constituting acquaintance could be partly a matter of its higher-order relational properties, i.e., the way in which it presents itself to consciousness and introspection^15^. Indeed, given that Russellian acquaintance is a function of the way in which the subject relates to her own mental states, it does not seem very revisionary to construe acquaintance in this way.

Something that may strike the reader about the above criteria for acquaintance is that they are all a function of extrinsic properties of perceptual intentionality, and not of its intrinsic properties. Phenomenal immediacy consists in the perceptual intentional state's not seeming to the subject to depend on awareness of something else; psychological noninferentiality is a matter of the state's not arising through an inferential process; direct causal relations to the object are straightforwardly extrinsic and relational; and sensory character is constituted both by the state's causal ties to sensations as well as its higher-order relational property of being typically phenomenally bound up with sensation, as far as consciousness and introspection are concerned. One may object that acquaintance should be wholly a matter of the intrinsic properties of a given state of awareness. It does not seem, however, that there is a principled reason to enforce such tight strictures on an account of what perceptual acquaintance consists in. Furthermore, it should be regarded as a rather considerable benefit that the extrinsic notions of acquaintance allow us to get some sort of independent theoretical traction on the idea of acquaintance, enabling us to get clearer on what we mean when we talk about being perceptually acquainted with objects and putting us in a better position to decide whether a particular case involves such acquaintance.

The above account is not intended to be complete. Nonetheless, the four proposed characteristics—actually five, considering that sensory character involves two different ways in which sensations leave their mark on perceptual intentionality—put us in a better position for understanding what acquaintance is, and for deciding on whether a given case constitutes acquaintance. To repeat, these conditions are not intended to be necessary and sufficient; it could be that none are necessary and none are individually sufficient, and that certain clusters are sufficient for acquaintance^16^. By way of vindicating these conditions, I will now argue that there are cases of acquired perception that fulfill them and that the cases that worry van Cleve (e.g., seeing his wife is home by virtue of seeing her keys on the table) do not. Perhaps some of Reid's examples of acquired perception don't involve perceptual acquaintance, but some do, and most importantly, acquired olfactory perception does.

The simplest attention to one's own experience, according to Reid, is sufficient to show that there are cases of acquired perception that are phenomenally immediate. Taking the example of a hearing the sound of a rolling coach as such, it would be very difficult (says Reid—and it seems hard to disagree) to deny that one's auditory awareness of the coach presents itself as unmediated by awareness of anything else (*IHM* 2.6, 38). It does not seem phenomenally to be the case that we first hear low-level auditory properties and then, in virtue of that perception, come separately to hear the sounds as emanating from horse feet. Reid says that we can hardly be convinced that our acquired perceptions are not innate (*EIP* 2.9). Indeed, with respect to the acquired perception of three-dimensional Euclidean figure, the primary reasons for positing a distinction between acquired and original visual perception are due to third-person conclusions about vision drawn from, among other things, facts about how painters simulate perceptions of three-dimensional shape with two-dimensional figures, and from the perceptual reports of patients whose congenital cataracts are removed. Phenomenally speaking, acquired perception is just as immediate a form of awareness of external objects as original perception. This fact is, for Reid, largely responsible for why the distinction between acquired and original perception is not to be found in ordinary language (*EIP* 2.9).

Whether cases of acquired perception are psychologically noninferential is a harder question to answer. On the one hand, we might tempted to say no, because it seems to involve first having an original perception; e.g., we originally just see visible figure, and then seeing visible figure causes us to see “real” or “tangible” figure. One could thus cast the psychological move from one perception to the next as a form of inference. On the other hand, the grounds for so casting it are unclear. It is doubtful, or at the very least open to debate, that the mere existence of a causal transition between contentful states is sufficient to constitute inference (Boghossian, in press). Even if it were true, it still seems that classic worries about the inferentiality of perceptual awareness arise not from mere worries about causal state transitions, but rather from the worry that the states that arise later in the causal chain are dependent in some richer sense on the earlier states. That is, the worry that the perception of 3D figure is mediated by inference is really a worry that perception of 3D figure is somehow derivative of perception of 2D figure; that our representation of 3D figure involves an inference according to some inferential scheme, *If there is 2D figure x, then there must be 3D figure y*. There is no reason to think perception of 3D figure by mature adults on Reid's account involves anything more robust than a brute-causal relation between perceptual states. In the absence of a reason to think that relation is inferential, and given its phenomenal immediacy, we can tentatively assume it to be noninferential^17^.

Acquired perception also involves direct causal relations to the environment. It is well-known that spelling out the necessary and sufficient conditions for the kind of direct causal relations that are required for veridical perception is tricky, given the existence of deviant causal chains (Chisholm, 1957; Grice, 1961; Dretske, 1981, 2003; Searle, 1983; Burge, 2010). In the typical 3D perception case, however (assuming there is some proper causal story to tell), the object's having the 3D shape it does clearly plays the crucial causal role in bringing about the veridical perception of that shape. Similarly for the size of the bell one hears, or the horse's hooves, and so on for many standard cases of Reidian acquired perception.

Typically, acquired perceptions have sensory character, with the major (though arguable) exception of the visual perception of shape^18^. With respect to the higher-order notion of sensory character, the case for the introspective inextricability of the sensory and intentional components of perceptual experience appears to be just as strong for cases of acquired perception as for original perception. To create an example, hearing a voice as the voice of a particular friend seems phenomenally intertwined with the qualitative character of the auditory sensations involved; and similarly, of course, for hearing the sounds of the coach grinding the cobblestones, and so on.

It is less clear whether acquired perceptions occupy the same sort of tight causal relations to sensations as original perceptions. It is an open question whether or not acquired perceptions always causally depend on prior token original perceptions that causally mediate sensation and acquired perception. Here is a reason to think that they do not. Reid simply does not have much of an account of the process of perceptual learning and how it enables acquired perception to occur. What little he does say is essentially that there is a constantly reinforced habituation process. Given just that meager constraint on how perceptual learning takes place, then it seems possible not only that acquired perception could occur on the onset of original perception, but also that the sensations themselves could give rise to an acquired perception immediately and concurrently with original perception^19^.

Suppose a given array of sensation-types, S, is innately hooked up to a certain original perception-type, P, and that perceptual learning enables one to have a token of the acquired perception-type, A, upon having a token of P. Abstracting from problem cases, every time a token of A occurs, a token of P occurs first; and every time P occurs, S occurs first. Then (again, limiting to the typical cases), it follows that any habituation or conditioning process that reinforces a connection between P and A will also reinforce a connection between S and A. S could therefore, at some point, simply give rise to A directly. There may be theoretical reasons why this could not happen—something about the mechanism that gives rise to A could preclude mere sensations from being causally sufficient, for example—but such reasons do not fall out of Reid's account. In the absence of a reason to think it cannot happen, then, since the bare bones of Reid's account imply that S could cause A directly, it seems that we can tentatively say that Reidian acquired perception can hook up to sensation directly. Even if that were not the case, and the connection between S and A must always be mediated by P, it seems that one could still consider that mediated relation a kind of tight causal connection that is sufficient for A to have sensory character and thus to be different from mere thought. Of course many thoughts have causal connections to sensations, but not the reliable causal structure of S—>P, P—>A. Acquired perceptions thus typically have sensory character in both the causal and higher-order senses.

### Testing the conditions

The above has hopefully sufficed to show that acquired perception fulfills the four (or five) conditions I have laid out for perceptual acquaintance. *Maybe so*, one might reply, *but then so much the worse for those conditions*. The reply may be that the conditions specified for perceptual acquaintance are too liberal, and that is the only reason why acquired perception looks like a species of perceptual acquaintance. Van Cleve's (2004, in press) helpful challenge to those who support the notion of acquired perception as perceptual acquaintance (e.g., Copenhaver, 2010) is to explain why his “seeing” that his wife is home by seeing her keys on the table does not fit the rubric for perception established by acquired perception. The spirit of the challenge is to show that construing acquired perception as a form of perceptual acquaintance doesn't just broaden the category of perception into triviality. This challenge is important because acquired perception has been invoked at crucial moments in the secondary literature on Reid to avoid Reid's theory of perception lapsing into incoherence or obvious falsity. For example, construing acquired perception as genuine perception is necessary to avoid saying that Reid's theory of the visual perception of the real shapes of objects amounts to indirect realism (Copenhaver, 2010; Quilty-Dunn, 2013), which is inconsistent with his fervent arguments against the idea that perception is indirect.

Fair enough, then: does van Cleve's example satisfy all the above conditions? It does not satisfy phenomenal immediacy. If van Cleve sees his wife's keys and “sees that” she is home, then it seems obvious that he can phenomenally distinguish two distinct acts of awareness and the asymmetric dependence relation that holds between them. One is aware of the set of keys, and aware that one's wife is home; furthermore, one is aware that the latter awareness is based on the former. Perhaps van Cleve would question that description of the phenomenology, but I find it difficult to see how it could be incorrect. If that's right, then the “perception” is not phenomenally immediate.

His awareness is also psychologically inferential. Unless the phenomenology is radically inadequate, then the inference drawn from the presence of the keys to van Cleve's wife being home is not only present but manifest in the experience. It also seems like the most obvious psychological interpretation of the situation is that he perceives the keys, thinks that if the keys are there then his wife is home, and draws the inference that his wife is home. In the absence of a reason to think otherwise, it seems from the first-person and third-person points of view that the case clearly involves inference.

Van Cleve's awareness of his wife is also not directly causally related to her. There is a causal connection between her and van Cleve's perception (viz., she put the keys there and they caused his perception) but it is not the kind that is unique to perception. This point relies on there being an account of the right kind of causal relation, which I cannot provide here. For one, however, when we are talking about visual perception, it could be argued that the direct causal relation must be carried out primarily through the medium of ambient light. There is no direct connection via ambient light between van Cleve and his wife, so it seems fair to say he is not visually acquainted with her^20^. By contrast, there is a direct connection through light between van Cleve and the 3D shape of the keys, so he may be visually acquainted with their 3D shape^21^.

Finally, van Cleve's intentional state directed toward his wife does not have sensory character. Focusing first on the higher-order notion, one could very likely pull apart the sensory qualitative character and the intentional state from the first-person with ease. The visual sensations would likely not seem inextricably wrapped up with one's awareness of a person who is absent from one's field of vision. Regarding the causal notion, the issue is a bit more difficult. On the one hand, there is a kind of causal connection between the sensations and van Cleve's awareness of his wife's being home. On the other hand, it seems that it is not the same kind that obtains between, for example, my auditory sensations, and my awareness of the C-minor chord in the song I hear. What exactly this difference consists in is hard to say, but that there is a difference in kind seems clear. Perhaps it consists in the subject's history of perceptual learning. If one studies music for years, one develops a very close causal tie between certain auditory sensation-types and awareness of certain musical properties; there does not seem to be a similar close causal tie, learned through normal processes of perceptual learning, for seeing one's wife to be home upon seeing her keys on the table.

Furthermore, with respect to ordinary acquired perception, I argued above that it seems open that the causal connection between sensations and acquired perceptions could come to obtain without being mediated by original perceptions. One might protest, for whatever reason, that this never actually happens. Even so, there is a difference between ordinary cases of acquired perception and the case with the keys on the table, which is that in the latter case, it seems impossible that it could happen. It is very difficult to see how the mere sensations could simply give rise to van Cleve's awareness of wife. It seems more natural to say that they give rise to such awareness only by first giving rise to an awareness of the keys themselves, leading to an inference that his wife is home.

If this discussion has been correct, then typical cases of Reidian acquired perception satisfy all the conditions laid out for perceptual acquaintance, and van Cleve's seeing that his wife is home by seeing her keys on the table satisfies none of them (certainly not all of them, in any case). These conditions thus fulfill the desiderata of enabling acquired perception to constitute perceptual acquaintance while ruling out the case of the keys on the table, i.e., avoiding triviality. It should be fair to appeal to them, then, in deciding whether acquired olfactory perception of the secondary quality of odor is possible. All we need to ask is whether acquired olfactory perception satisfies most or all of the conditions for perceptual acquaintance.

## Olfactory perceptual acquaintance

It is important to keep in mind the important characteristics of acquired olfactory perception mentioned in Two Solutions. The olfactory conceptions that form constituent elements of acquired olfactory perception are, in terms of their semantic properties, relative notions of whatever quality is causally responsible for generating certain types of sensations. Nonetheless, though that is Reid's semantic account of such conceptions, when they figure in an occurrent olfactory perception, they need not represent the odor by explicitly appealing to its relation to the occurrent sensation. There is a principled difference between, on the one hand, representing one's occurrent olfactory sensation O^*^ followed by representing there to be a causal relation between it and some external quality O, and on the other hand, having O^*^ and then simply representing external quality O, without the latter representation being mediated by representing O^*^ and without explicitly representing the relation between the tokens of O and O^*^.

Both ways of becoming aware of O do involve relations to O^*^, because both are caused by O^*^, and because the perceiver's notion of O is a notion of some external quality that bears a certain causal relation to sensations of type O^*^. But the first kind of awareness involves inferring there to be some quality O that causes *this* token O^*^ sensation; the second kind simply involves representing there to be O, and the notion of O happens to be a notion of the kind of thing that causes sensations of type O^*^. The second kind of awareness does not involve an occurrently mediated form of awareness. It is both phenomenally immediate and psychologically noninferential, thus satisfying those two conditions for perceptual acquaintance.

The acquired olfactory perception of O also stands in a direct causal relation to the instance of O itself in the environment. There is particularly good reason to say so if, as suggested in the previous section, the crucial causal connection for a given sensory modality involves the medium that is proprietary to that modality. Certainly typical olfactory perception will involve such a connection to the olfactory qualities of external objects. Reid describes the medium of olfaction as involving “effluvia” (i.e., airborne particles) emanating from objects, so olfactory perceptual states are causally related to olfactory qualities of external objects via that medium.

Olfactory sensations are also quite intimately bound up with the intentionality of olfactory perception, and thus imbue that intentionality with sensory character in both senses mentioned in the previous section. As far as our ordinary consciousness and even reflective introspective awareness of olfactory perception is concerned, for Reid, we do not separate the qualitative character of the sensations from the perception of the external olfactory quality. According to at least the early Reid in *IHM*, our terms for the qualitative aspects of odors are better understood to refer to the qualitative characters of olfactory sensations than to external olfactory properties, so closely are olfactory sensation and perceptual intentionality bound up in consciousness and introspection (*IHM* 2.2, 27). With respect to the causal construal of sensory character, acquired olfactory perceptual states are closely keyed to olfactory sensations. This condition is only met once adequate perceptual learning has taken place, but once it has, then the relevant acquired perceptions are caused directly by the sensations^22^.

By all the standards set above for perceptual acquaintance, acquired olfactory perception plainly constitutes such acquaintance.

## Conclusion

I have tried to resolve the tension between Reid's theory of perception and his account of our conception of odors. It has long been noted in the secondary literature that acquired perception is required, but problems still lingered. Two points are really central to preserving the coherence of Reid's theory. First, it is necessary to make clear the distinction between the semantics of our conceptions of odors involving relations to sensation, and our occurrent perceptual awareness of them being subjectively predicated on relations to occurrent sensations. Second, it is necessary to argue that some instances of acquired perception, including olfactory perception, do constitute perceptual acquaintance such as to block van Cleve's negative arguments.

According to the resulting interpretation, our notions of odors are based on inferring their causal relations to our sensations, but they can be perceived noninferentially through acquired perceptual acquaintance. Reid's theory of olfactory perception is therefore coherent, and for contemporary philosophers, perhaps attractive. A Reidian account allows one to explain the qualitative character of olfactory experience in terms of perceptual sensation, and to explain the intentional or representational aspect of such experience without having to adopt anything more robust than a dispositional (or dispositional-base) account of odors. It also provides, given the interpretation advanced above, a relatively tidy explanation of the acquisition of our capacity to represent dispositional properties such as odors in perception.

There is an odd, and oddly popular, caricature of Reid prevalent among present-day philosophers. According to this caricature, Reid thinks that all phenomenal character in perception is due to nonintentional sensations, and that the intentional component of perception involves no phenomenal character at all. Clare Batty, for instance, writes, “If we take it that Reidian sensations are one and the same as what we now think of as experiences, then Reid himself also held that olfactory experiences are purely sensational” (Batty, 2010a, p. 520; see also Siegel, 2011, p. 21; Smith, 2002, p. 70). If the terms of the present-day debate were explained to him, it seems far more likely that Reid would say perceptual experience is not merely a matter of sensation but also of the intentional component of perception—which, I have argued, constitutes perceptual acquaintance. It would be hard for him to deny, for example, that visual perceptual intentionality affects the way things *look* to the subject, which seems sufficient for its affecting visual phenomenology. By the same token, representing there to be a certain odor in an object or in the environment will affect the way things seem to the subject in her olfactory perceptual experience.

Reid's account of olfaction thus allows for both a representational account of our experiences of odors as properties of objects or environments, and also for an account of the qualitative character of olfactory perceptual experience, while requiring no more substantial an ontological commitment than to dispositional properties (or their bases). Reid's account is also consistent with a representational account that locates odors in the object (which is how he sometimes talks), or with one that “locates” them simply as immanent in one's immediate environment (see *IHM* 6.8, 99; see Batty, 2010a for discussion of the relative merits of these views).

Finally, one need not be wedded to Reid's dual-component (sensation and belief) view of perception to make use of his account. One could instead say that our notion of odor is relative to our notion of the qualitative properties of olfactory experiences (without regarding those properties to be instantiated in states called sensations) and that the intentional or representational contents of those experiences can come to incorporate acquired representations of odors as dispositions or dispositional bases, without supposing the intentional/representational component to involve belief, as Reid does. Anyone who endorses a distinction between the qualitative and intentional aspects of perceptual states might thus be able to employ Reidian ideas—which should be a particularly attractive option for theorists who also take odors and other secondary qualities to be dispositional properties. A Reidian theory of olfactory perception should, for all these reasons, be considered a live option in contemporary debates on olfaction and secondary qualities generally.

### Conflict of interest statement

The author declares that the research was conducted in the absence of any commercial or financial relationships that could be construed as a potential conflict of interest.

## Footnotes

^1^The term “odor” is sometimes used to refer to qualities of external objects, and ptsometimes to refer to particular clouds of airborne particles or locations (see Batty, 2010a,b). Following what I take Reid's standard usage to be, I typically use the term to refer to qualities of objects; hopefully, not much hangs on this terminological point.

^2^Olfaction is of special interest in understanding Reid's account of secondary-quality perception, and not simply because Reid takes it to be the proper initial area of inquiry into perception. Vision and tactition involve perception of both primary and secondary qualities, and Reid says very little about gustation aside from likening it to olfaction. Gustation is also arguably wrapped up with tactition in a way that olfaction is not. Reid's discussion about audition, though useful in understanding his account of secondary-quality perception, is also largely about language and Reid's doctrine of natural signs. Olfaction is thus useful because, on Reid's account, it appears to be a sense purely dedicated to a particular kind of secondary quality. Furthermore, I will argue that Reidian olfactory perception is entirely acquired and in no part innate (see Two Solutions and Olfactory Perceptual Acquaintance), whereas this is false of vision and tactition and not obviously the case for audition—for example, Reid seems to believe in an innate faculty for the auditory perception of musical qualities (*IHM* 4.2, 50). Understanding Reid's account of olfactory perception is useful, therefore, because it is purely a matter of the acquired perception of a particular kind of secondary quality. Given its simplicity as well as Reid's special interest in it, olfaction is uniquely suited to provide an understanding of Reidian secondary-quality perception.

^3^When I refer to the intentional component of perception, or to perceptual intentionality, I mean to refer to the conception–belief element of perceptual awareness. When I refer to perceptual awareness, I generally mean the complex of sensation and intentionality.

^4^See, e.g., the letter to the earl of Findlater and Seafield that opens *IHM* for Reid's announcement of his intentions in this regard (*IHM* Dedication, 3–6).

^5^The “base” view seems more popular. For articulations and defenses of that view, see (Lehrer, 1989; Wolterstorff, 2001; McKitrick, 2002); for the dispositional view, see Van Cleve (2011). For what it's worth, I think the base view is likely the correct interpretation of Reid, but since I am here only concerned with the perception of secondary qualities and not with their ontology, nothing hereafter should hang on it.

^6^See (Wolterstorff, 2001; Buras, 2002; Smith, 2002; Van Cleve, 2002, 2004). For defense of Reid's direct realism, see Copenhaver (2004), Quilty-Dunn (2013).

^7^See (Smith, 2002), Chapter 2, for an interesting critical discussion of Reid and Sellars, and of the similarities between the two.

^8^This led Reid to develop a fascinating and ingenious sketch of a non-Euclidean geometry in 1764 (*IHM* 6.9), several decades before such projects were incorporated into mainstream Western mathematics [Daniels (1974); see Yaffe (2002) for a thorough discussion]. It was not well-recognized as fascinating or ingenious at the time. Joseph Priestley, in his critical examination of Reid's *IHM*, remarked, “I do not remember to have seen a more egregious piece of solemn trifling than the chapter which our author calls the “Geometry of Visibles” (Priestley, 1775, p. 99–100).

^9^Following a somewhat standard notation, I use a letter to denote a type of quality of external objects, and the same letter followed by an asterisk to denote the type of sensation that corresponds to it.

^10^Of course, whether I need to have the sensations first is a separate question, and has no bearing on the inference/reference point. A token sensation can causally precede a perceptual intentional state without being referred to or rendering the intentional component of perception inferential; indeed, that is how Reidian perception typically functions.

^11^For a primer on the psychology of perceptual learning, see Kellman and Garrigan (2009).

^12^Batty argues that olfaction represents odors (conceived of as particulars in the ambient environment, rather than qualities of objects) as being “here” [2010a, p. 524–525; see also Batty (2010b), Richardson (2013)].

^13^Lycan, similarly, says there is no analog of stereopsis in olfaction, despite the presence of two nostrils and olfactory bulbs, which is a mere “superfluity” (Lycan, 2000, 287n12). This is probably incorrect. Porter et al. (2007) found that the distance between the nostrils facilitates different odor sampling in each nostril; furthermore, they showed that scent-tracking in humans is significantly worse when odors are equally distributed between both nostrils.

^14^As has been noted (see, e.g., Byrne, 2005), the conceptual/nonconceptual distinction could apply to the kind of content or the kind of state involved. I hope here to avoid making any claims on either side.

^15^Consciousness, for Reid, as a mental operation, is noninferential higher-order awareness. For discussion of whether Reid thought that higher-order views offered the right theory of what it is for a state to be conscious —which is a separate question—see Copenhaver (2007). This is not the place, however, to offer an interpretation of Reid's theory of consciousness (if he held one at all).

^16^For instance, given that the visual perception of shape does not constitutively involve sensation for Reid [though see Yaffe (2003a)], the points about sensory character might not apply. In the case of auditory perception of the size of a bell, on the other hand, the sensory character seems crucial to its constituting acquaintance.

^17^In Quilty-Dunn (2013), I argued that the perception of 3D figure could be considered immediate noninferential perception if it was the sort of awareness that is proprietary to perception (and not mere thought), echoing (Van Cleve, 2004). This was vague and unexplored; the notion of perceptual acquaintance outlined here should provide a clearer and more substantive account of what makes a certain form of awareness count as *perceptual* awareness.

^18^See Yaffe (2003a), Falkenstein and Grandi (2003), Yaffe (2003b) for an extended discussion of whether the visual perception of shape constitutively involves sensation.

^19^See Goldstone (1998) for an overview of various mechanisms of perceptual learning that could underwrite such a process.

^20^Perhaps there could be visual perception, with the same sensory qualitative character and perceptual contents, via a distinct medium (e.g., using a prosthetic eye that relies on sonar, or to borrow from Daniels, 1974, sensitivity to gravitational fields). These issues present a thorny set of problems. Nonetheless, in the case we are considering, there is nothing to replace the ambient light, so such problems should not arise.

^21^Copenhaver claims that a certain amount of “practical engagement” with a perceptible property that is “prevalent in one's environment” (Copenhaver, 2010, p. 305) can facilitate acquired perception. This condition is problematic, however, since it is compatible with the case currently being examined becoming, through the right sort of practical engagement, an instance of perceptual acquaintance (Ibid.). It seems to me that it is impossible to be perceptually acquainted with someone who is not in one's field of vision. The condition of a causal connection via a proprietary causal medium—e.g., ambient light—suffices to rule out such a case. In any case, practical engagement is arguably successful in giving rise to acquired perception only insofar as it facilitates the right kind of perceptual learning, which might be facilitated through non-practical modes of interaction with the relevant perceptible property.

^22^In fact, the issue about whether the causal connection between sensation and acquired perception must be mediated by an original perception does not even arise on the interpretation advanced here, according to which there is no original perception in olfaction.
